# An Overview of Recent Developments in Improving the Photocatalytic Activity of TiO_2_-Based Materials for the Treatment of Indoor Air and Bacterial Inactivation

**DOI:** 10.3390/ma16062246

**Published:** 2023-03-10

**Authors:** Achraf Amir Assadi, Oussama Baaloudj, Lotfi Khezami, Naoufel Ben Hamadi, Lotfi Mouni, Aymen Amine Assadi, Achraf Ghorbal

**Affiliations:** 1 Center for Research on Microelectronics and Nanotechnology, CRMN Sousse Techno Park, Sahloul BP 334, Sousse 4054, Tunisia; 2 Research Unit Advanced Materials, Applied Mechanics, Innovative Processes and Environment, Higher Institute of Applied Sciences and Technology of Gabes (ISSAT), University of Gabes, Gabes 6029, Tunisia; 3 Laboratory of Reaction Engineering, Faculty of Mechanical Engineering and Process Engineering, Université des Sciences et de la Technologie Houari Boumediene, BP 32, Algiers 16111, Algeria; 4 Laboratory of Advanced Materials for Energy and Environment, Université du Québec à Trois-Rivières (UQTR), 3351, Boul. des Forges, C.P. 500, Trois-Rivières, QC G9A 5H7, Canada; 5 Chemistry Department, College of Science, Imam Mohammad Ibn Saud Islamic University (IMSIU), Riyadh 11432, Saudi Arabia; 6 Laboratoire de Gestion et Valorisation des Ressources Naturelles et Assurance Qualité, Faculté SNVST, Université Bouira, Bouira 10000, Algeria; 7 École Nationale Supérieure de Chimie de Rennes (ENSCR), Université de Rennes, UMR CNRS 6226, 11 Allée de Beaulieu, 35700 Rennes, France

**Keywords:** semiconductor, photocatalysis, indoor air treatment, volatile organic compounds, microorganism

## Abstract

Indoor air quality has become a significant public health concern. The low cost and high efficiency of photocatalytic technology make it a natural choice for achieving deep air purification. Photocatalysis procedures have been widely investigated for environmental remediation, particularly for air treatment. Several semiconductors, such as TiO_2_, have been used for photocatalytic purposes as catalysts, and they have earned a lot of interest in the last few years owing to their outstanding features. In this context, this review has collected and discussed recent studies on advances in improving the photocatalytic activity of TiO_2_-based materials for indoor air treatment and bacterial inactivation. In addition, it has elucidated the properties of some widely used TiO_2_-based catalysts and their advantages in the photocatalytic process as well as improved photocatalytic activity using doping and heterojunction techniques. Current publications about various combined catalysts have been summarized and reviewed to emphasize the significance of combining catalysts to increase air treatment efficiency. Besides, this paper summarized works that used these catalysts to remove volatile organic compounds (VOCs) and microorganisms. Moreover, the reaction mechanism has been described and summarized based on literature to comprehend further pollutant elimination and microorganism inactivation using photocatalysis. This review concludes with a general opinion and an outlook on potential future research topics, including viral disinfection and other hazardous gases.

## 1. Introduction

Air pollution and the degradation of air quality are becoming severe issues to deal with, but these notions often remain abstract and complex to surround or identify [1]. The contamination of food and drink raises a lot of interest since they are linked to vital daily elements for everyone [2]. Understanding how airborne particles can affect food and beverage quality is the first step to understanding how air filtration systems can address this issue [3]. Therefore, it is natural that the agri-food sector has a significant challenge in protecting its employees and processes against harmful atmospheric pollutants [4].

The primary pollutants confronted in indoor air include carbon monoxide (CO), micro-organisms (fungi, bacteria, and viruses), nitrogen oxides (NOx), and a multitude of varieties of volatile organic compounds (VOCs) [5]. Given that in France, agri-food companies represent 15.3% of manufacturing industries with more than 17,647 companies [6], it is therefore essential and urgent to employ the purification system technology more effectively [7,8].

Numerous developing and encouraging technologies currently supply a solution to this issue [9,10]. Among them, heterogeneous photocatalysis in visible light proves its interest in compounds’ degradation and/or mineralization [11,12]. However, these technologies do not make it possible to effectively guarantee constant purification over time of the microorganisms without the need for frequent maintenance operations due to their excessive bulk [13,14]. Advanced Oxidation Processes (AOPs) are processes that produce highly oxidizing species such as hydroxyl radicals (^•^OH) and other reactive oxygen species (ROS), including the anion superoxide radical (^•^O_2_^−^) and hydrogen peroxide (H_2_O_2_) capable of degrading target pollutants present in effluents [15,16]. The semiconductor material TiO_2_ is considered a reference photocatalyst and an antibacterial agent due to its physicochemical properties [11,17,18]. However, TiO_2_ has a wide bandgap, which limits its practical application in environmental remediation under visible light irradiation, including a wide range of the solar spectrum [16,19]. Many strategies have been implemented to overcome this concern, such as doping TiO_2_ with metallic or non-metallic elements [20] and coupling with other semiconductors [21,22,23] to increase their absorption in the visible and improve the lifetime of electron-hole pairs [24]. It is possible to improve the redox process of pollutant degradation by doping TiO_2_ with a metal oxide, which produces photoexcited charge carriers [25].

Indoor air quality has emerged as a significant public health problem. Photocatalytic technology is a natural solution for deep air filtration due to its low cost and excellent efficiency. Photocatalysis methods have been extensively researched for environmental remediation, notably for air treatment. Several semiconductors, such as TiO_2_, have been used as photocatalytic catalysts, and they have gained a lot of attention in recent years due to their remarkable properties. For indoor air purification and bacterial inactivation, this review has compiled and evaluated current findings on improvements in the photocatalytic activity of TiO_2_-based photocatalytic materials. The characteristics of various popular TiO_2_-based catalysts and their benefits in the photocatalytic process have also been clarified, as well as how doping and heterojunction approaches might increase photocatalytic activity. Recent articles regarding diverse combined catalysts have been summarized and examined to underline the relevance of combining catalysts to boost efficiency. The studies that employ these catalysts to remove microorganisms and volatile organic compounds (VOCs) were also covered in this publication. Based on the literature, the reaction mechanism has also been defined and summarized to understand better pollutant removal and microorganism inactivation utilizing photocatalysis. Finally, this review’s conclusion includes a summary and prognosis on prospective future study areas, such as viral disinfection and other dangerous gases. To our knowledge, there are few studies on the catalytic activity of alternative materials for indoor air treatment by eliminating both pollutants types, microorganisms, and VOCs.

## 2. Photocatalysis and Mass Transfer

Heterogeneous photocatalytic oxidation (HPO) is one of the active investigations in environmental treatment and purification [26,27,28]. It is widely applied in air pollution treatment, especially volatile organic compounds [29,30]. The resourceful technology is reserved for decomposing gaseous contaminants by employing photocatalysts under UV or solar light free of additional energy expenses [13,31].

### 2.1. Principle of Photocatalysis

**Figure 1:**
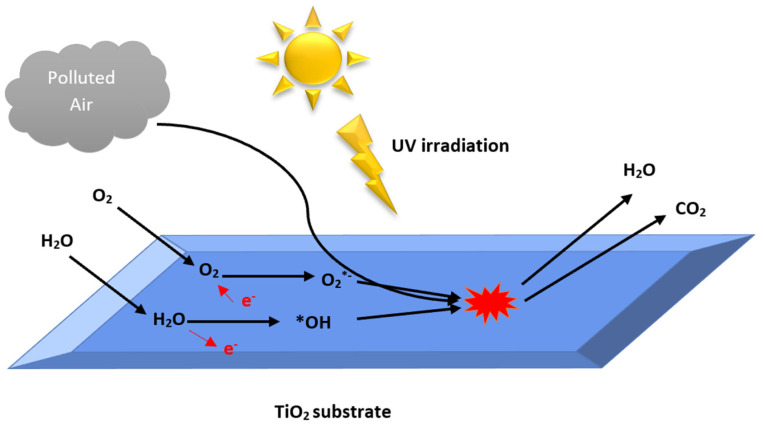
Polluted air photocatalysis treatment by TiO_2_.

Photocatalysis is generally described as the process of employing light (UV or visible light) to activate a substrate (such as a semiconductor photocatalyst) so that photo-reaction can be accelerated or facilitated with the catalyst remaining unconsumed [5]. The process can be divided into five steps (Figure 1):

(1) Transfer the reactants to the air phase.
(2) Adsorption of the reactants on the surface of the catalyst.
(3) Reaction in the adsorbed phase.
(3.1) Absorption of a photon by the catalyst.
(3.2) Generation of the electron-hole pairs.
(3.3) Separation of the pair.
(4) The oxidation and reduction with the adsorbed substrate.
(5) Desorption of the intermediate product.

**Figure 2:**
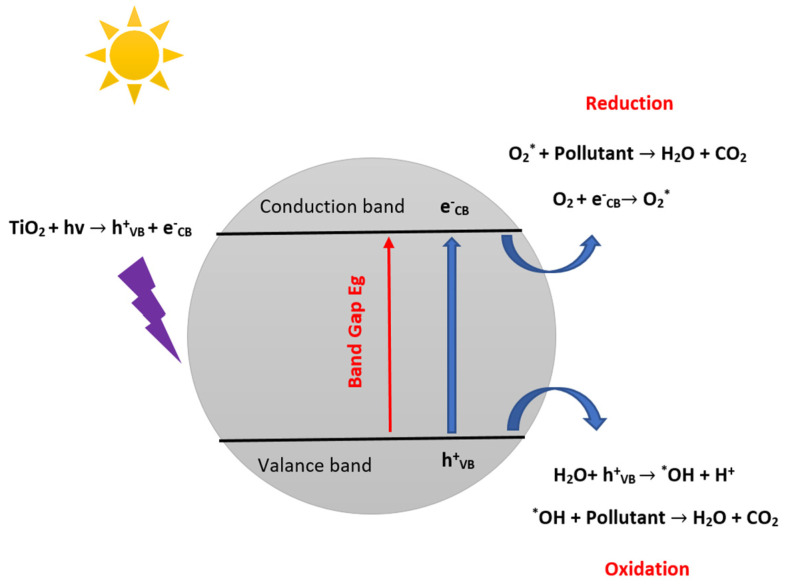
Schematic illustration of the photocatalytic reaction mechanism.

Among these five steps, the photocatalytic reaction is of crucial significance. It is initiated by the electron’s excitation from the filled valence band (V_B_) to the empty conduction band (C_B_) of the photocatalyst when the energy carried by the absorbed photon equals or exceeds the band gap of the photocatalyst (Figure 2). In addition, the reaction results in the creation of a negative electron in the C_B_ and a positive hole in the V_B_ is called an electron-hole pair [32,33,34]. The positive hole oxidizes the hydroxide ion to yield hydroxyl radical (^•^OH), a potent oxidant of organic pollutants. The photo-excited electron is reduced to form the superoxide radical anion (O_2_^•−^). These radicals are keys to the degradation of organic compounds [35].

### 2.2. Development of Heterogeneous Photocatalytic Oxidation

Among all semiconductors, Titanium dioxide (TiO_2_)-based materials have received particular attention in the photocatalysis field for their light absorption ability and high-efficiency treatment for both water and air; it was discovered by Fujishima and Honda in 1972 [36]. According to previous investigations, TiO_2_-based photocatalysts also provide the advantages of high stability, availability, nontoxicity, excellent photoactivity, and low cost [35]. The photocatalysis of TiO_2_ depends on variables such as specific surface area, crystallinity and surface hydroxyl groups of the TiO_2_ [37]. This particular material has relatively polar surfaces that allow easy adsorption of hydrophilic pollutants. Nevertheless, titanium dioxide (TiO_2_)-based materials have rapid recombination of electron-hole pairs, which, to some degree, suppresses the reaction efficiency [38,39].

Moreover, the band gap of materials is wide (3.0–3.2 eV), so the reaction is only activated with the irradiation of ultraviolet; hence the utilization of visible light irradiation is limited [14,40]. In order to improve the activity of photocatalysts under solar or artificial light at a lower energy cost and under more economic conditions, several strategies and investigations have been carried out to enhance the performance of TiO_2_ [41]_._ These strategies include chemical modification, dye sensitization, and coupling with other semiconductor materials by introducing impurity atoms into pure TiO_2_ to change electron-hole pairs concentrations in TiO_2_ [35].

Metal doping is a method in which traces of foreign elements are introduced within the crystal lattice, and researchers widely use this strategy to reduce the band gap of titanium dioxide-based materials [42]. Noble metallic metals such as Ag, Au, Pt, and Pd have been researched extensively for years because of their properties and contribution to visible light absorption [5].

Ag is of particular interest as it acts as an electron trap and leads to retard the recombination of the electron-hole pair through the improvement of the transfer of interfacial charge [43]. Yi et al. studied a composite of Ag–AgI–TiO_2_/CNFs; the Ag and I (Iodine) oxidation generated the reactive oxygen species (ROS) in the visible light range [44], doping TiO_2_ with Ag and I increase its light range, increasing the photocatalytic activity. Yangfeng Chen et al. proposed a composite of heterostructured g-C_3_N_4_/Ag/TiO_2_ microspheres by using the properties of Ag to delay the recombination of electron-hole pairs [45]. Apart from Ag, other metallic oxides can be composited with titanium dioxide to make the photo-reaction work under visible light. For example, halogens (X: Cl, Br, or I) bound to Bismuth oxide to form BiO_X_, as a new class of promising catalyst has also drawn significant attention due to their attractive physicochemical characteristics, such as unique micro/nanostructures, bandgaps, optical and electrical properties and many other physicochemical characteristics [46,47,48,49]. Wendong Zhang et al. [49] found that the nanoplate BiOBr was highly efficient under visible light for NO photoreduction. Those promising catalysts (halogens) can be used as heterostructured photocatalysts with TiO_2_ in order to enhance their photocatalytic activity. Actually, There is a lot of work done in heterogeneous photocatalysts with TiO_2_, such as TiO_2_/Ag [50], TiO_2_/SiO_2_ [51], TiO_2_/Fe_2_O_3_ [52,53]_,_ TiO_2_/Graphene [54] which have been shown to improve the photocatalytic performance of TiO_2_, especially in the degradation of organic pollutants. These are only a few examples of TiO_2_-based heterostructured photocatalysts. TiO_2_ may be mixed with a variety of different substances to improve its photocatalytic activity. Aguilera-Ruiz also stated that cuprous oxide (Cu_2_O), a visible-light-driven photocatalyst, has a band gap of about 2.07 eV [55]. Meanwhile, the conduction and valence band boundaries of BiVO_4_ are located at 0.11 V and 2.65 V NHE. Thus, the composite Cu_2_O/BiVO_4_ has a promising photocatalytic performance under visible light [56]. Those two interesting materials, CuO and BiVO_4_, can be used as the heterojunction or heterostructure to enhance the photocatalytic activity of the TiO_2_-based catalysts. Moreover, it has been shown that Ag- V- and Fe-doped TiO2 achieved by various routes are very efficient in the oxidation of VOCs (butyl acetate, hexane or gaseous toluene) [57].

Non-metal doping is another strategy established to increase titanium dioxide’s activity under solar or visible light. This technique takes advantage of the possible electronic transition from the induced new electronic states above TiO_2_ V_B_ (2p or 3p orbitals of the dopant) to TiO_2_ C_B_ (3d orbitals of Ti). Several researchers reported that the doped photocatalyst activity increases after non-metal doping, as the electronic structure has been modified to extend the absorption of the photocatalyst into the visible-light region [57]. Various studies have shown non-metal doping of TiO_2_, such as Nitrogen-doped TiO_2_ [58], carbon-doped TiO_2_ [59,60,61], sulfur-doped TiO_2_ [62,63], boron-doped TiO_2_ [64] and phosphorus-doped TiO_2_ [65]. Vaiano et al. studied recyclable visible-light active N-doped TiO_2_ photocatalysts coated on glass spheres using a simple sol-gel method. They obtained excellent photocatalytic activity with visible light irradiation [66].

### 2.3. Reactors and Configurations

The configuration of reactors for air treatment is a critical element in the efficiency of the process. It should promote effective contact between the catalyst and the photons on the one hand and between the catalyst and the pollutants on the other. Care must also be taken to limit pressure drops. This part will present different continuous-flow photoreactors used in the laboratory or on an industrial scale.

Usually, this type of reactor is made up of a perforated plate placed at the inlet to ensure the homogeneity of the airflow. The central box contains two fixing devices for the photocatalytic support on one hand and a UV lamp on the other. In this configuration, the polluted air, driven by a fan, passes through the photocatalytic support. Another reactor configuration is based on using porous monolithic supports with varying thicknesses. The structure is based on the successive use of several UV lamps and monolithic "honeycomb" type photocatalytic media. The lamps irradiate the front and back sides of the monolithic supports.

The photocatalytic medium is placed against the reactor’s internal wall and irradiated by a lamp set in a central tube. The particularity of this type of pilot is that the distance between the two plates or the diameters carrying the media is variable, which makes it possible to test the effect of the gap on the performance of the process.

Flat and cylindrical configurations:

A schematic representation of this rectangular configuration is used in the works of Assadi et his co-workers [3,5,17]. This reactor is formed by a chamber containing two glass plates at a variable distance. Each plate carries the photocatalytic media. Lamps are positioned along the length of the reactor at an equal distance in the inter-plate space [3,5,17].

The reactor is formed by two cylindrical tubes. The catalyst is installed on the inner wall of the outer cylinder. A UV lamp is installed in the inner tube in order to have uniform radiation from the catalytic surface. The gaseous effluent circulates between the outer tube’s inner wall and the inner tube’s outer wall. Note, To demonstrate the effect of material transfer, the diameter of the inner tube is studied to vary the thickness of the gas film [3,5,17].

**Figure 3:**
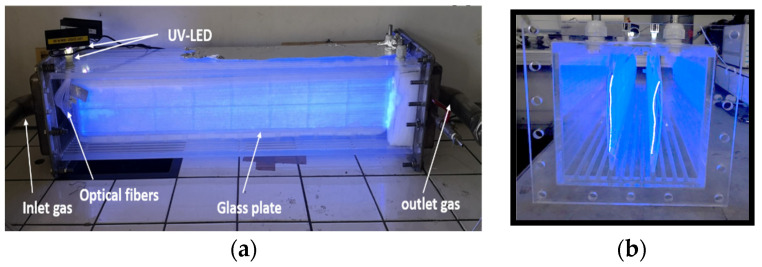
(**a**) Images of a photocatalytic reactor based on optical fibers, (**b**) of a side view (Range of processed flow: from 5 at 20 m^3^/h with concentrations varying from 5 to 50 mg/m^3^) [5].

Another planar configuration is based on surface-degraded fiber optic sheets on which titanium dioxide has been deposited. The latter replaced the bulky UV lamps. Optical fibers are used to activate the catalyst and further optimize the supply of UV radiation compared to lamps. This configuration will make it possible to inactivate the pollutants while offering compactness of the solution, i.e., lower pressure drops and easy handling in use. Figure 3 shows images of a fiber optic photocatalytic reactor and a fiber optic shee [3,5,17].

## 3. Volatile Organic Compounds (VOCs)

Volatile organic compounds are substances containing organic carbon which vaporize at significant rates [67,68]. They are the second-most widespread and various emissions classes after particulates [7]. Besides, we can state that as an approximate rule, VOCs are the organic liquids or solids whose vapor pressures at room temperature exceed 0.01 psi (=0.0007 atm) with atmospheric boiling points equal to or less than 480 °F estimated at 101.3 kPa, i.e., standard atmospheric pressure. Among hundreds of VOCs that have been qualitatively identified in the indoor environment, the main compounds are alkanes, alkenes, carboxylic acids and alcohols, esters, and aromatics [69]. Organic compounds are primarily found in home items such as wax, varnishes, and paints. All these chemicals can emit organic byproducts when utilized and stored in a non-controlled method. Studies have revealed that levels of numerous organic chemicals indoors are 2 to 5 times greater than outside. Therefore, with a specific level of time exposure, these organic compounds may have short or long term adverse health effects such as headaches, eye and respiratory tract irritation and even cancers [70].

VOCs in the atmosphere or the environment are relatively at low concentrations; hence, they are detectable based on interactions between the sensor component and the organic compounds. In addition, ventilation is also a conventional dilution method. Still, it is not firmly recommended in current practice because of its limitation on outdoor air quality (OAQ) and energy consumption [11,17,18]. Accordingly, researchers are still developing technologies and efficient approaches to meet IAQ standards and reduce energy costs to avail a secure, healthful, livable environment. During their research, Abidi and his collaborators studied the elimination of chloroform CHCl_3_ by using different catalysts and analyzing the removal efficiency under several initial concentrations of each catalyst type supported on polyester under certain conditions [5]. Many works have demonstrated the ability of some TiO_2_-based photocatalysts to remove VOCs from the air due to their high photocatalytic activity and stability [71]. Tobaldi et al., 2021 have reported that TiO_2_-graphene oxide composites exhibit enhanced photocatalytic activity for the removal of various VOCs, such as benzene, toluene, and formaldehyde [72]. Another work has shown that TiO_2_-carbon nanotube composite photocatalysts have efficient and improved photocatalytic activity for the removal of various VOCs, such as xylene and toluene [73]. The efficiency of the TiO_2_ can be enhanced in its photocatalytic activity for the elimination of VOCs in the air by doping it with metal oxides such as ZnO, Fe_2_O_3_, and WO_3_ [74], as this doping can increase the surface area and prevent electron-hole recombination. Table 1 summarizes several recent studies on the removal of VOCs using TiO_2_-metal.

## 4. Microorganism Inactivation and Reactional Mechanisms

**Figure 4:**
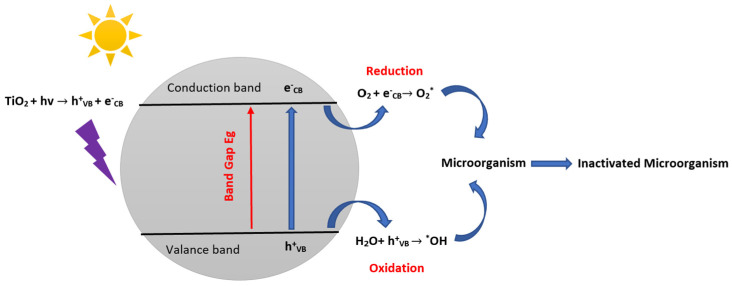
Schematic illustration of the antibacterial photocatalytic mechanisms of TiO_2_ (inspired from ref. [89]).

Understanding the mechanism of the bactericidal effect action of semiconductors is fundamental to improving its activity and, in particular, involves the analysis of the targets of TiO_2_ at the bacterial level [88]. TiO_2_ is a multifunctional photocatalyst that may be utilized to render microorganisms inactive [61]. The following steps are involved in the overall process for the inactivation of microorganisms using TiO_2_. Step 1: TiO_2_ is exposed to irradiation and undergoes a photocatalytic reaction that produces ROS like hydroxyl radicals (*OH) and superoxide radicals (O_2_*^−^). Step 2: ROS is formed in the photocatalytic reaction and interacts with bacterial cells and membranes and damaging DNA, proteins, and lipids. In addition, ROS can combine with water molecules to form more ROS, such as H_2_O_2_. Damages and harm caused by ROS interactions lead then to step 3 inactivation of the microorganism, in which the cell of the microorganism dies. The general microorganisms’ photocatalytic inactivation mechanisms of TiO_2_ can be summarized by the following equations and Figure 4:

TiO_2_ + hv → TiO_2_ (C_B_ e^−^) + TiO_2_ (V_B_ h^+^) (1)

O_2_ + e^−^ → O_2_^−*^ (2)

H_2_O + h^+^ → H^+^ + *OH (3)

*OH + O_2_^−*^ + Microorganism → Inactivated Microorganism (4)

**Figure 5:**
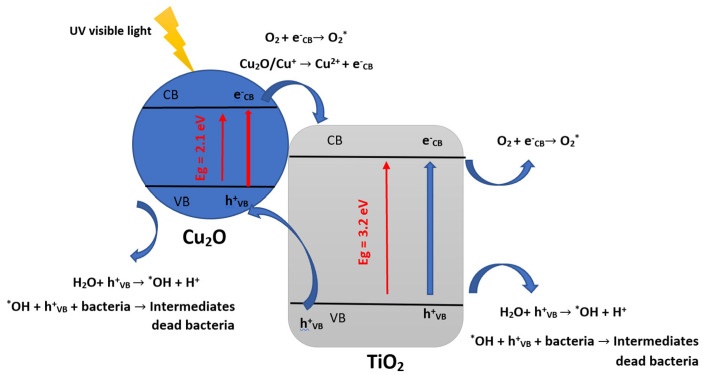
The main antibacterial photocatalytic mechanisms of TiO_2_ with Cu_2_O (inspired from refs [78,92,93]).

Overall, because TiO_2_ is ecologically neutral and doesn’t produce toxic byproducts, using it as a photocatalyst to inactivate bacteria presents a viable substitute for conventional disinfection techniques that involve chemicals or heat [90]. The effectiveness of TiO_2_-based photocatalysis, however, is dependent on several variables, including the characteristics of the TiO_2_, the strength and wavelength of the light source, and the kind and quantity of bacteria present [91]. The inorganic semiconductors doping or adding a co-catalyst, such as TiO_2_, with metals such as Cu, mainly accelerates bacterial inactivation kinetics [76,92]. Different reactions will likely be generated when the copper oxides are in contact with the catalyst’s surface [76,92]. Indeed, CuO and Cu_2_O are spawned when there is an interaction between copper and O_2_ (air) under light irradiation. Cu_x_O is found in two forms (CuO and Cu_2_O) and exhibits the Cu(+I) and Cu(+II) oxidation states, of which the main form that interacts with bacteria and VOCs is Cu_2_O, thus generating electrons at the level of the conduction band; Cu_2_O (C_B_ e^−^) and holes in the valence band; Cu_2_O (V_B_ h^+^) [78,92,93]. Under simulated sunlight, Cu_2_O (C_B_ e^−^) enters a reduction reaction with TiO_2_ to reduce Ti^4+^ to Ti^3+^ and yields Cu(+I) at the V_B_ h^+^ level, which may lead to bacterial inactivation and/or VOCs to form CO_2_, H_2_O, N, S and inactivated bacteria [17,76,92]. The main antibacterial photocatalytic mechanisms of TiO_2_ with Cu_2_O suggested by previous research papers cited above can be summarized by the following equations and Figure 5 [78,92,93]:

Cu_2_O + hv → Cu_2_O (C_B_ e^−^) + Cu_2_O (V_B_ h^+^) (5)

Cu_2_O (C_B_ e^−^) +TiO_2_ → TiO_2_^−^ ou (Ti^3+^) + Cu_2_O (6)

TiO_2_^−^ + O_2_ → TiO_2_ +O_2_^−*^ (7)

°O_2_^−^ + h^+^ → H_2_O* (8)

H_2_O* + h^+^ + e^−^_cb_ → H_2_O_2_ (9)

H_2_O_2_ + e^−^_cb_ → OH + *OH (10)

Cu_2_O (V_B_ h^+^) + bacteria → CO_2_ and H_2_O (11)

h^+^ + Bacteria → Inactivated Bacteria (12)

H_2_O_2_ + Bacteria → Inactivated Bacteria (13)

*OH + Bacteria → Inactivated Bacteria(14)

The inactivation of bacteria cells can occur by many processes during the photocatalytic reactions, either the rupture of the cell membrane (Membrane disruption), the cell wall (Exposed cellular components), or the attack of the cells by ROS [19]. Where high levels of oxidative stress may be produced by ROS, which interacts with bacterial cells effectively and kills them by destroying the cell wall and a variety of bacterial cell components such as protein, lipids, carbohydrates, DNA, and amino acids [94]. Furthermore, when photocatalyst particles are deposited at the surface of bacterial cells, they can interact with them via diffusion and endocytosis mechanisms, which induce the destruction of membrane proteins or cell membranes owing to the phenomena of member permeability [19]. Moreover, both catalysts and generated ROS can interfere with the movement of electrons within the cell microorganisms, loss of protein motive force, depletion of intracellular ATP production with DNA replication disintegration, and intracellular outflow resulting in bacteria cell inactivation [95].

The hydroxyl radicals (*OH) produced on the surface of copper (Cu^+^) in contact with H_2_O with the holes generated at the level of V_B_ h^+^ is the primary ROS involved in bacterial inactivation [29,96]. Cu_2_O exhibits high bacterial inactivation capacity when light irradiation stimulates electron transfer between copper and bacterial cells and produces reactive oxygen species (ROS), resulting in bacterial cell inactivation [78,97,98]. Abidi et al. investigated the effects of Cu_x_O amounts at different sputtering times on the TiO_2_-Polyester (PES) photocatalyst in the inactivation of microorganisms [17]. For sputtering intensities ranging from 20 to 80 A, it was regarded that the Cu_x_O/TiO_2_–PES catalyst sputtered at 80 A; the total inactivation of the bacteria was obtained after an hour of exposure to indoor light. Copper oxide showed high antibacterial activity, and the intrinsic activity of Cu(+I) can be enhanced by UV-vis illumination [17].

Additionally, Ag-NP is an excellent material used to improve the photocatalytic inactivation of microorganisms using TiO_2_, which has recently been proven in previous works [76]. Ag particles could inactivate bacteria as Ag-NP is an essential factor that controls and regulates antimicrobial activity [92].On contact of Ag with TiO_2_ under light irradiation, either Ag(0), Ag(+I), or Ag(+II) are yielded. The release of these different forms of Ag in contact with *Escherichia coli* induces bacterial inactivation [99]. The Ag used for TiO_2_-NT decoration showed +1 and +2 oxidation states (Ag^+^ and Ag^2+^) [78].

In its metallic state, silver is oxidized in the air (O_2_), breeding Ag_2_O; this substance yields Ag^+^ ions. This 4-electron process can be outlined by the following two equations [99]:

4 Ag^0^ + O_2_ → 2 Ag_2_O (15)

2 Ag_2_O + 4H^+^ → 4 Ag^+^ + 2 H_2_O (16)

Ag_2_O is at the origin of the inactivation of bacteria when it generates the production of reactive oxygen species in contact with TiO_2_-NTs. While Ag_2_O is in contact with TiO_2_ as a semiconductor, electrons (e^−^) are photo-generated by the semiconductor under the action of bandgap radiation as indicated by the chemical reaction (Equation (20)) and photo-generated holes (h^+^) react with H_2_O (Equation (18)) to yield hydroxyl radicals (OH°) [76,92]:

Ag_2_O + e^−^ → 2 Ag^+^ + ½ O_2_^−^ (17)

h^+^ + H_2_O → *OH + H^+^ (18)

2H_2_O + O_2_ + 2e^−^ → 2 *OH + 2 OH^−^ (19)

e^−^ + O_2_ → O_2_*^−^ (20)

**Figure 6:**
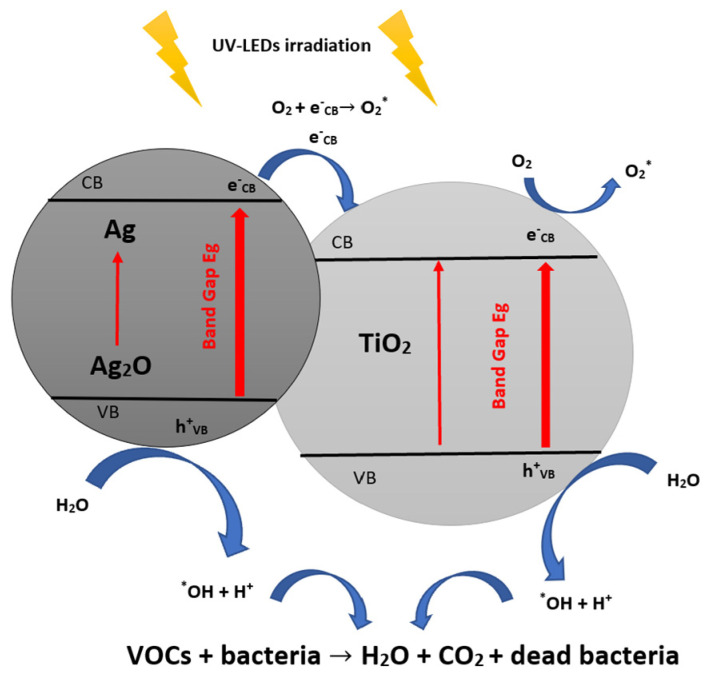
The main antibacterial photocatalytic mechanisms of TiO_2_ with Ag_2_O (inspired from ref. [76]).

The suggested bacterial inactivation mechanism with Ag/TiO_2_ under light can be recapitulated in the following equations [76] and Figure 6:

Ag_2_O + hv → Ag_2_O (C_B_ e^−^) + Ag_2_O (V_B_ h^+^) (21)

Ag_2_O (e^−^ + h^+^) + TiO_2_ → Ag_2_O (V_B_ h^+^) + TiO_2_ (C_B_ e^−^) (22)

TiO_2_ (C_B_ e^−^) + O_2_ → TiO_2_ + O_2_ (23)

2 e^−^ + O_2_ + 2H^+^ → H_2_O_2_ (24)

H_2_O_2_ + O_2_^−^ → *OH + OH^−^ + O_2_ (25)

Ag_2_O (V_B_ h^+^) + Bacteria → Inactivated Bacteria (26)

H_2_O_2_ + Bacteria → Inactivated Bacteria (27)

*OH + Bacteria → Inactivated Bacteria(28)

*OH + VOCs → CO_2_ + H_2_O (29)

It is well known that the reaction of oxygen radicals in the cell causes its death [96]. Furthermore, different bacteria have different membrane structures [100]. For example, Gram − bacteria have peptidoglycan of the wall less thick than Gram + bacteria, which have an additional outer membrane composed of a double layer of lipids. This finding is in chains of different catalytic reactions and further disinfection efficiencies [101]. Accordingly, other bacteria’s survival rates will differ under identical disinfection conditions [102].

The most critical mechanism in antibacterial activity is cell membrane damage. Oxidative stress generated by ROS is a second mechanism involved in antibacterial activity [103,104,105]. This stress inhibits DNA replication, protein synthesis, and cellular metabolism, causing cell death [106]. In order to demonstrate the effect of ROS on cell death, a study was conducted in the absence and presence of L-cysteine, a natural antioxidant, with *E. coli* bacteria. Indeed, growth was inhibited by Cu-TiO_2_/GF with an efficiency of 79.4% in the absence of L-Cysteine compared to 65.1% in its presence. Similarly, Ag-TiO_2_/GF, where the efficiency was 100% without the antioxidant and diminished to 84.7% in its presence [92]. The experiment is conducted on the following bacteria: *E. coli* and *Staphylococcus aureus* (*S. aureus*) on copper-doped TiO_2_/GF and silver-doped TiO_2_/GF synthesized by sol-gel method, and at different relative humidities (Table 2).

Overall, silver-doped TiO_2_/GF performed best on both bacteria, followed closely by copper TiO_2_/GF and [107] then TiO_2_/GF alone. The yield was better at a relative humidity of 60% than 80%. They were significantly lower at 40% humidity. *E. coli* is eliminated reasonably than *S. aureus* since the latter is a Gram + bacterium with a more complex wall [76,92].

## 5. Conclusions and Outlook

Recent studies on photocatalysis for indoor air purification and bacterial inactivation have shown promising results. Even though a variety of photocatalysts are available, TiO_2_-based materials are the most effective or, at the very least, effective option for practical and financial reasons. This review has collected and covered recent research that has improved the photocatalytic activity of materials based on TiO_2_ for VOC degradation in indoor air and bacterial inactivation. Coupling TiO_2_ materials with other methods has been increasingly explored. This paper also reviewed the literature on the material aspects of photocatalysis based on AgxO/TiO_2_ and CuxO/TiO_2_ to treat air-containing chemical and biological pollution. A bibliographical synthesis of the type of catalyst and the operating conditions was detailed concerning the decontamination of VOCs. Moreover, the different types of microorganisms treated by TiO_2_-based photocatalysts have been listed. In-depth explanations of the reaction mechanisms for photocatalytic degradation and inactivation have been provided. As a look ahead to future research, we believe more study and testing are needed to clarify and comprehend the benefits of TiO_2_-based materials on photocatalytic applications. There are only a few works on the combined treatment of chemical and biological pollution using photocatalysis at the same time. The investigations do not consider evaluating removal or if mineralization is complete, which is an essential criterion because it can generate more harmful intermediates than the pollutant. Finally, experiments in real cases of air pollution, such as hospital air pollution, are required to apply this process.

## Figures and Tables

**Table 1:** List of some studies on using TiO_2_-metal for VOCs removal.

Target Pollutants	Reactors	Catalyst	Radical Species	Operating Conditions	Degradation Performance	Formed Products (Intermediate and Final)	Ref.
Propionic acid (PPA) and benzene (BENZ)	annular reactor + dielectric barrier discharge (DBD)	SiO_2_-TiO_2_ + UV	°OH, CH_3_ CH_2_°	SiO_2_ = 6.5 g m^−2^ et TiO_2_ = 6.5 g m^−2^ performance lamp UV-A (80 W/10) output intensity (25 W/m^2^) Odor inlet concentrations 0.068 to 0.405 mmol m^−3^, Q = 2 at 6 m^3^ h^−1^ relative Humidity: 5 to 90%, T = 20 °C	RE tested alone: 55% (APP) et 40% (BENZ) RE of mixture: 50% for APP and 30% for BENZ RE combined process: 60% for a voltage equal to 9 kV RE of mixture gaseous effluent (5% HR): 50% APP et 50% BENZ	BENZ: CO_2_ dominating CO weak, O_3_, CH_3_CH_2_OOH instable → Alcool + Aldéhyde → CO_2_ PPA: CO_2_, ethanoic acid (CH_3_CH_2_OOH), ethanol (CH_3_CH_2_OH), aldehyde (CH_3_CHO), H_2_O, O_2_	[75]
Butane-2,3-dione and Heptane-2-one	Continuous Planar Reactor	TiO_2_, TiO_2_-Cu et TiO_2_-Ag	^•^OH, O_2_°^−^	Q = 1–12 m^3^ h^−1^ concentration of COV= 5–20 mg.m^−3^ Humidity level = 5–70%, under UV-A light oxidation.	RE of TiO_2_ alone: 63% RE of TiO_2_-Ag: 46% RE of TiO_2_-Cu: 52%	acetone (C_3_H_5_O) propionic acid (C_3_H_6_O_2_) butanoic acid (C_4_H_8_O_2_) pentanoic acid (C_5_H_10_O_2_) acetic acid (C_2_H_4_O_2_) acetaldehyde (C_2_H_4_O) formic acid (HCOH) carbon dioxide (CO_2_) and H_2_O	[76]
Acetone and toluene	Surface DBD discharge	Pt/TiO_2_ and MnO2/CuO_2_/Al_2_O_3_	NS	Concentration: 0.2 ppm flow rate: 38.42 m^3^/h	100% toluene destruction of toluene at 0.2 ppm and 100% acetone destruction at 0.46 ppm	NS	[77]
Butane-2,3-dione (BUT) + *E. coli*	spherical batch reactor	Cu_2_O/TiO_2_ and TiO_2_-Ag	^•^OH, HO_2_° and O_2_°^−^	Concentration: 4.4 g/m_3_ T = 50 at 100 °C λ = 380–420 nm, under UV–vis light irradiation.	99.7% *E. coli* inactivation and 100% VOC degradation within 60 min and 25 min with TiO_2_-Ag for simultaneous treatment	CO_2_, H_2_O	[78]
methyl ethyl ketone (MEK) or 2-butanone	annular reactor	TiO_2_ (fiberglass + Ahlström support)	^•^OH, O_2_^−^°, °H_2_C-CH_3_, °CH_3_, H_3_C-C°=O, °H_2_C-CO-CH_2_-CH_3_	MEK concentration on glass fibers: 1.51 mg/L MEK concentration on Ahlström: 1.75 mg/L HR glass fibers: 0.11–3.94 mW/cm^2^ HR Ahlström: 0.12–2.53 mW/cm^2^ T = 30 °C and 20 vol.% O_2,_ under UV light source.	Deposition of TiO_2_ on glass fibers leads to 10% degradation of MEK for 1.5 mg/L. TiO_2_ Ahlström leads to the elimination of 40% of MEK for 1.5 mg/L.	acetaldehyde (C_2_H_4_O) ethane (C_2_H_6_) methane (CH_4_) methanol (CH_3_OH) acetone (C_3_H_6_O) methyl formate (C_2_H_4_O_2_) carbon dioxide (CO_2_) and H_2_O	[79]
Acetone	annular reactor	TiO_2_ (fiberglass + Ahlström support)	°CH_3_, ^•^OH, H_2_C°-COOH, H_3_C-°C=O	Concentration: 14.9 ng/L and 66.0 ng/L light power: 0.21 to 3.94 mW/cm^2^ T = 30 °C, 20 vol.% O_2_ Volume flow: 150 to 300 mL/min, under UV light.	90% of Acetone conversion has been obtained for low initial concentrations with TiO_2_ photocatalyst deposited on fiberglass for simultaneous treatment	acetaldehyde (C_2_H_4_O) methyl alcohol (CH_3_OH) isopropyl alcohol (C_3_H_8_O) methyl ethyl ketone (C_4_H_8_O) acetic acid (CH3 COOH) mesityl oxide (C_6_H_10_O) diacetone-alcohol (C_6_H_12_O_2_)	[80]
Benzene	the outer surface of the rectangular SiC ceramic membrane	Pt/SiC@Al_2_O_3_	NS	0.176% by mass of Pt	90% reduction at 215 °C with a space velocity of 6000 mg^−1^ h^−1^	CO_2_, H_2_O	[81]
n-butanol and acetic acid	fixed-bed tubular reactor	Pt/CeO_2_-AlO_3_	NS	1000 ppm of COV T = 50–350 °C 0, 7, 15, 23 et 51% by weight of CeO_2_	100% reduction for n-butanol at T < 250 °C 50 or 90% reduction for a reduction of 80 or 20 °C.	Butanal (C_4_H_8_O) methanol (CH_4_OH) propanol (C_3_H_8_O) isopropanol (C_3_H_8_O) formaldehyde (HCOH) propanal (C_3_H_6_O) carbon dioxide (CO_2_)	[82]
Formaldehyde	organic glass reactor	Pt/AlOOH/, Pt/AlOOH-c, Pt/c-Al_2_O_3_ and Pt/TiO_2_	NS	HCHO concentration: 127 ppm for adsorption and 139 ppm for catalytic oxidation, fan: 5 W T: 35 °C HR: 25% oxidation time: 51 min.	Pt/AlOOH > Pt/AlOOH-c > Pt/c-Al_2_O_3_ > Pt/TiO_2_	surface formate carbon dioxide (CO_2_) water (H_2_O)	[83]
Formaldehyde	fixed-bed quartz flow reactor	Ag/TiO_2_, Ag/Al_2_O_3_ et Ag/CeO_2_	NS	Concentration: 110 ppm T = 35 to 125 °C Debit: 100 mL min^−1^, under light containing ultraviolet.	Ag/TiO_2_ > Ag/Al_2_O_3_ > Ag/CeO_2_ 100% HCHO conversion with Ag/TiO_2_ at T = 95°C	carbon dioxide (CO_2_) another carbon-containing compound	[84]
Formaldehyde	NS	Pt/TiO_2_, Rh/TiO_2_, Pd/TiO_2_, Au/TiO_2_ (noble metals/TiO_2_)	NS	Concentration: 100 ppm 1% noble metals/TiO_2_ O_2_ 20 vol.% Debit: 50 cm^3^ min^−1^ T: 20 °C GHSV: 5000 h^−1^	Pt/TiO_2_ ≫ Rh/TiO_2_ > Pd/TiO_2_ > Au/TiO_2_	carbon dioxide (CO_2_)carbon monoxyde (CO); water (H_2_O)	[85]
Dimethyl disulfide (DMDS)	Continuous Flow Quartz Tubular Reactor	(Au + Pd)/TiO_2_, Au/MCM-41, (AU + Rh)/MCM and Au/TiO_2_, Pd/TiO_2_	NS	3%Pd/TiO_2_ and 1%Au/TiO_2_(1%Au + 3%Pd)/TiO_2_ gas flow: 42,000 h^−1^ Temperature: 20–320 °C	Au/TiO_2_ and Au-Pd/TiO_2_ effectively remove DMDS for T < 155 °C Au/MCM-41 less effective in DMDS eliminating	methanol (CH_3_OH) ethanol (C_2_H_6_O) methyl mercaptan (CH_3_SH) ethyl mercaptan (CH_3_SCH_3_) hydrogen sulfur (H_2_S) carbon dioxide (CO_2_) carbon monoxide (CO) sulfur dioxide (SO_2_) water (H_2_O)	[86]
toluene + m-xylene + ethyl acetate or acetone	fixed-bed Quartz Continuous Flow Microreactor (ICP-AES)	0.91 wt.% Au_0_._48_ Pd/α-MnO_2_et α-MnO_2_	α-, β- et γ-oxygène	1% (Au-Pd) Mixing flow: 17 mL/min concentration: 1000 ppm + O_2_ + N_2_ (solid) molar ratio COV/O_2_ = 1/400 SV (space velocity) = 40,000 mL (g h) T = 320 °C	0.91 wt.% Au 0.48 Pd/α-MnO_2_ > α-MnO_2_	carbon dioxide (CO_2_)water (H_2_O)	[69]
Isovaleraldehyde	continuous annular plasma reactor DBD combined photocatalysis	TiO_2_	^•^OH, O_2_^•−^	concentration: 75 to 200 mg m^−3^ Debit: 2 m^3^ h^−1^ HR: 5% T: 20 °C I: 20 W m^−2^ SE: 17 J L^−1^, under UV light.	NS	propanoic acid (CH_3_CH_2_COOH) propanone (CH_3_COCH_3_) ethanoic acid (CH_3_COOH) carbon dioxide (CO_2_) carbon monoxide (CO) ozone (O_3_)	[87]
Benzene	New UV-LED frontal flow photocatalytic reactor	TiO_2_ deposed on luminous textiles	OH°, O_2_°^−^	concentration: 100 to 200 mg m^−3^ Debit: 1 m^3^ h^−1^ HR: 5 to 80% T: 20 °C		CO_2_ and H_2_O	[72]

**Table 2:** List of some studies on using TiO_2_-metal for bacterial inactivation.

Bio Contaminants	Reactor	Catalyst	Operations Parameters	Performance	Ref.
*E. coli*	Petri dishes	TiO_2_-NT and Ag-TiO_2_-NTs	Concentration: 4 × 10^6^ UFC/mL volume: 100 mL diameter TiO_2_: 100 nm at 70V diameter Ag: 8 nm	TiO_2_: reduction of 1.6 log with 180 min Ag/TiO_2_: reduction of 99.99% after 90 min	[107]
*P. aeruginosa*	Glass fiber tissue (GFT)	Poroux TiO_2_TiO_2_ pur (TiO_2_-PEG) and TiO_2_-Ag	Concentration: 10^3^ UFC/mL TiO_2_ pur: 14.7 nm TiO_2_-Ag-PEG:16.6 nm TiO_2_-Ag: 25.3 nm, under UV light.	TiO_2_-1Ag: 100% of inactivation after 10 min TiO_2_ poroux: 57% TiO_2_-PEG: 93%	[108]
*E. coli* K12	Agar matrix surface + blueberry skin + calyx	UV-TiO_2_& UV alone	Initial bacterial populations: 7 log CFU/g UV-Photocatalysis (4.5 mW/cm^2^) UV alone (6.0 mW/cm^2^). TiO_2_-coated quartz tubes (38 cm length, 24.5 mm outer diameter, thickness 0.7–0.9 mm.	4.5 log CFU/g for UV alone and 5.3 log CFU/g for UV-TiO_2_ in 30 s. 3.4 log and 4.6 log CFU/g, respectively, UV alone and UV-TiO_2_ for the first 30 s. 4.0 log and 5.2 log CFU/g, respectively, UV alone and photocatalysis.	[109]
*S. aureus. P. aeruginosa* and *E. coli*	LB agar plates	TiO_2_-Ag (TiO_2_ (calcinated at 300 °C) (CB300) at (500 °C_)_ (CB500) et TiO_2_ (not calcinated) (CB))	Concentration: 10 µL with 10^9^ UFC/mL 5%w of TiO_2_	TiO_2_ (calcined 300 °C)-Ag: reduces bacterial growth by 95%, i.e., 1.05 × 10^8^ CFU/mL with UV. TiO_2_ (calcined 500 °C) without Ag: reduces bacterial growth by 30% with UV. TiO_2_ (calcined at 300 °C) without Ag: reduces growth by 75%.	[110]
*E. coli*	Planar reactor	TiO_2_, TiO_2_-Ag and TiO_2_-Cu deposed on optical fibers	Initial bacterial populations: 2.4 × 10^7^ UFC/mL. The core of optical fibers is constructed of polymethyl methacrylate resin with a mean diameter of 480 m and coated with 10 m of a thick fluorinated polymer, under UVA-LEDs (365 nm, UVA-LED intensity = 1.5 W m^−2^).	3 log of removal with TiO_2_/Ag and TiO_2_/Cu	[76]
*S. aureus* CCM 3955 & *S. aureus* CCM 3953 (Gram+) *E. coli* & *P. aeruginosa* (Gram−)	Disposable plates	Ag NPs	Initial bacterial populations: from 10^5^ to 10^6^ UFC/mL, Particle size from 40 to 60 nm, Temperature 35 °C.	Higher activity at 7 ppm against *P. aeruginosa*. NP Ag synthesized based on AgNO_3_: considerable antibacterial activity at 14 and 29 ppm (82.49% inactivation). NP Ag synthesized based on AgNO_3_ and citrate: 88.56 inactivations.	[111]
*E. coli*	Batch reactor	Cu_2_O-NPs/TiO_2_-NTs catalyst	Initial bacterial populations: from 10^6^ to 10^7^ UFC/mL. Under visible light irradiation (380–720) nm. Temperature 37 °C.	Bacterial inactivation rate of 98% and a concomitant 99.7% VOC removal within 60 min and 25 min	[78]
